# A Personalized Approach Bias Modification Smartphone App (“SWiPE”) to Reduce Alcohol Use: Open-Label Feasibility, Acceptability, and Preliminary Effectiveness Study

**DOI:** 10.2196/31353

**Published:** 2021-12-10

**Authors:** Victoria Manning, Hugh Piercy, Joshua Benjamin Bernard Garfield, Stuart Gregory Clark, Mah Noor Andrabi, Dan Ian Lubman

**Affiliations:** 1 Monash Addiction Research Centre Eastern Health Clinical School Monash University Melbourne Australia; 2 Turning Point Eastern Health Melbourne Australia; 3 School of Psychological Sciences Monash University Melbourne Australia; 4 School of Medicine Monash University Melbourne Australia

**Keywords:** alcohol, hazardous alcohol use, alcohol use disorder, approach bias modification, cognitive bias modification, smartphone app, ehealth, mobile phone app, mhealth, digital health

## Abstract

**Background:**

Approach bias modification (ApBM), a computerized cognitive intervention that trains people to “avoid” alcohol-related images and “approach” nonalcohol images, reduces the likelihood of relapse when administered during residential alcohol treatment. However, most individuals experiencing alcohol problems do not require, do not seek, or have difficulty accessing residential treatment. Smartphone-delivered ApBM could offer an easily accessible intervention to reduce alcohol consumption that can be personalized (eg, allowing selection of personally relevant alcohol and positive nonalcohol training images) and gamified to optimize engagement.

**Objective:**

We examined the feasibility, acceptability, and preliminary effectiveness of “SWiPE,” a gamified, personalized alcohol ApBM smartphone app, and explored alcohol consumption and craving outcomes in people drinking at hazardous levels or above (Alcohol Use Disorders Identification Test [AUDIT] score ≥8) who wanted to reduce their alcohol use.

**Methods:**

In this open-label trial, frequency and quantity of alcohol consumption, alcohol dependence severity, and craving were measured prior to participants downloading SWiPE. Participants (n=1309) were instructed to complete at least 2 sessions per week for 4 weeks. Recruitment and completion rates were indicators of feasibility. Functionality, aesthetics, and quality ratings were indicators of acceptability. Participants were prompted to report frequency and quantity of alcohol consumption weekly during training and 1 month after training. They completed measures of craving and dependence after 4 weeks of training.

**Results:**

We recruited 1309 participants (mean age 47.0, SD 10.0 years; 758/1309, 57.9% female; mean AUDIT score 21.8, SD 6.5) over 6 months. Participants completed a median of 5 sessions (IQR 2-9); 31.2% (409/1309) completed ≥8 sessions; and 34.8% (455/1309) completed the posttraining survey. Mean Mobile Application Rating Scale scores indicated good acceptability for functionality and aesthetics and fair acceptability for subjective quality. Among those who completed the posttraining assessment, mean past-week drinking days reduced from 5.1 (SD 2.0) pre-training to 4.2 (SD 2.3) in week 4 (*t*_454_=7.87; *P*<.001), and mean past-week standard drinks reduced from 32.8 (SD 22.1) to 24.7 (SD 20.1; *t*_454_=8.58; *P*<.001). Mean Craving Experience Questionnaire frequency scores reduced from 4.5 (SD 2.0) to 2.8 (SD 1.8; *t*_435_=19.39; *P*<.001). Severity of Dependence scores reduced from 7.7 (SD 3.0) to 6.0 (SD 3.2; *t*_435_=12.44; *P*<.001). For the 19.4% (254/1309) of participants who completed a 1-month follow-up, mean past-week drinking days and standard drinks were 3.9 (SD 2.5) and 23.9 (SD 20.7), respectively, both significantly lower than at baseline (*P*<.001).

**Conclusions:**

The findings suggest SWiPE is feasible and acceptable and may be effective at reducing alcohol consumption and craving in a predominantly nontreatment-seeking sample of adult Australians drinking at hazardous levels. SWiPE’s efficacy, relative to a control condition, now needs establishing in a randomized controlled trial. Smartphone-delivered personalized ApBM could be a highly scalable, widely accessible support tool for reducing alcohol use.

**Trial Registration:**

Australian New Zealand Clinical Trials Registry ACTRN12620000638932; https://www.anzctr.org.au/Trial/Registration/TrialReview.aspx?ACTRN=12620000638932p

**International Registered Report Identifier (IRRID):**

RR2-10.2196/21278

## Introduction

Alcohol remains the most widely used drug globally [1,2] and is a leading cause of injury, chronic disease, and mortality (contributing to 3 million deaths per year), accounting for 5.1% of the global burden of disease [3]. In 2019, 16.8% of Australians aged over 14 years reported drinking above the recommended national guidelines of 14 standard drinks per week, while 25% drank more than the recommended 4 standard drinks in 1 day at least monthly [4]. Unsurprisingly, alcohol remains a drug of concern for almost 50% of all treatment contacts in Australia’s publicly funded addiction treatment services [5], although recent modelling suggests this system likely only meets 27% to 56% of the potential demand for treatment [6]. Barriers to seeking alcohol treatment include limited treatment availability, limited time, poor knowledge of treatment options, fear of stigma, geographical distance, privacy concerns, or a belief that the individual can address their alcohol problems without professional help [7].

With approximately 90% of Australians now owning a smartphone [8], development of app interventions offers the potential to overcome many of these barriers. Although hundreds of apps claim to help people reduce alcohol use, very few have been evaluated. A systematic review of studies completed by the end of 2019 identified only 12 trials of alcohol reduction apps designed for adults [9]. Only 5 of these studies were randomized controlled trials (RCTs), of which only 2 demonstrated efficacy. The clarity of these findings is limited because of the disparate interventions used by different apps (including normative feedback, self-monitoring, psychoeducation, action planning, goal setting, problem-solving skills, and identifying or managing triggers and cravings) that target various mechanisms, although broadly speaking, most of them aimed to strengthen “reflective” cognitive processes used to control behavior.

According to the “incentive-sensitization” model [10], repeated use of addictive drugs sensitizes the neural reward system, strengthening the attention-grabbing and motivational properties of alcohol and its associated cues [11] (such as physical and social contexts, sights, sounds, scents), leading to “attentional bias” [12] toward these cues and cue-induced craving [13]. This also leads to the development of “approach bias” (the automatic, impulsive action tendency to approach alcohol-related cues) [12]. Craving [14,15], approach bias [16], and attention bias [17] have all been found to predict heavy alcohol use or relapse. Since alcohol-related cues are ubiquitous and nearly impossible to avoid, in Australia (like many other countries), the craving and cognitive bias that can be elicited by these cues pose a serious challenge for people seeking to reduce or cease their drinking.

Alcohol approach bias can be reduced, or even reversed, through a form of computerized “brain training” known as approach bias modification (ApBM) [18-21]. In ApBM, individuals are repeatedly presented with alcohol-related images, to which they must make an “avoidance” movement (eg, “pushing away” images using a joystick), and nonalcohol-related images, to which they must make an “approach movement” (eg, “pulling” the image toward oneself using a joystick). This trains individuals to automatically “avoid” alcohol-related cues. Several RCTs have shown that, when delivered as an adjunctive intervention during residential treatment for alcohol use disorders (AUD), 4 to 12 sessions of ApBM (typically lasting 10-15 minutes per session) can reduce likelihood of posttreatment relapse [18-20,22,23].

Although residential treatment settings are appropriate for people with severe AUD [24], there is a much larger population of people with less severe alcohol use problems that adversely impact health and quality of life [25,26] who want to reduce or cease drinking. Smartphone-delivered ApBM may be particularly advantageous for this broader population. Using a smartphone, people could complete ApBM training sessions at times and in places that are most convenient for them (eg, at times or in situations where they are vulnerable to experiencing heightened craving). Generalization of training effects may be enhanced by completing ApBM in naturalistic environments rather than in clinical settings.

Thus far, we are aware of only 2 previous studies examining ApBM smartphone apps. In the United Kingdom, Crane et al [27] tested apps containing various combinations of 5 different modules (including an ApBM module) among people drinking at hazardous levels. Despite initially reporting that combining ApBM with normative feedback reduced participants’ weekly alcohol consumption [27], they later reported a lack of evidence for efficacy after re-analyzing outcomes with a larger sample [28]. In the Netherlands, Laurens et al [29] tested an ApBM app with people who were concerned about, or wished to reduce, their drinking. Over a 3-week training period, weekly alcohol consumption declined by a mean of 7.2 standard drinks, relative to pretraining [29]. Participant feedback was generally positive, though participants noted the monotony and repetitiveness of the ApBM training, suggesting that game-like features could make it more engaging. Participants also noted the lack of personalization (participants were all trained using the same standardized set of beverage images and participants).

In our research on treatment seekers for AUD [19,22,30], we have observed that participants tend to drink a limited range of beverages. Thus, use of a standard image set of beverages for all participants reduces the relevance of the training to individuals (eg, being repeatedly trained to avoid images of beer may have little impact for someone who only drinks wine). Since approach bias is the product of associative learning [31], it is likely to be specific to stimuli resembling the drinks frequently consumed by an individual. Designing ApBM tasks where individuals can use their own “personalized” images is therefore likely to be more engaging and more “potent” at reducing approach bias. Personalization can be easily implemented in smartphones by allowing participants to incorporate their own photos of the beverages they most wish to “avoid.”

It is not only the “avoidance” stimuli that could be personalized. In almost all alcohol ApBM research to date [18-20,22,23,27,29,30], participants have been systematically trained to approach nonalcoholic beverages. However, these images are likely to be monotonous and of relatively little personal relevance to patients [29]. Recently, we have begun exploring the use of images representing positive, personal goals or personally preferred healthy sources of pleasure (eg, images symbolizing friends, family, social connection, pets, exercise, financial gain) as “approach” stimuli in ApBM training for substance use disorders [32,33]. This responds to recommendations that these should align with patients’ goals for behavioral change or offer alternative strategies to manage cravings [34-37]. In this way, personalized ApBM can simultaneously be used to weaken motivations to drink and reinforce positive goals, which may further increase its overall therapeutic benefit. In a smartphone app, people could use their own photographs of friends, family, or hobbies as approach stimuli, making the positive “approach” stimuli highly tailored to the individual’s motives for reducing their alcohol use. Including gamified aspects in the task may also improve engagement even further, enhancing completion rates and thereby further enhancing efficacy.

Drawing on the aforementioned body of research, we recently developed “SWiPE,” a novel, world-first, personalized alcohol ApBM app. We aimed to test its feasibility, acceptability, and preliminary effectiveness in an open-label, single-group pilot study in people reporting hazardous alcohol use (ie, a score of 8 or more on the Alcohol Use Disorders Identification Test [AUDIT], a commonly used AUD screening tool [38]) recruited from the general community. In addition, we collected data regarding drinking, alcohol craving, and alcohol dependence severity outcomes following training, to inform the design of a future RCT of this app. As previously stated in the published protocol [33], we hypothesized that:

1. We would recruit at least 500 participants within 6 months of launching the app and that at least 60% of participants would complete 8 sessions of ABM, supporting its feasibility.
2. Mean ratings of SWiPE would be greater than 3 on the “functionality,” “aesthetics,” and “app subjective quality” subscales of the user version of the Mobile Acceptability Rating Scale (uMARS) [39], demonstrating adequate acceptability.
3. There would be statistically significant decreases in number of standard drinks per week, number of days on which alcohol was used in the past 7 days, alcohol craving, and Severity of Dependence Scale (SDS) [40] scores at the end of the 4-week intervention, relative to pretraining scores, suggesting its potential effectiveness.
4. There would be “dose-response” relationships, whereby the degree of reduction between the pretraining and 4-week assessments in measures of alcohol consumption, craving, and dependence severity will be related to the number of ApBM sessions completed over this period (ie, more sessions will be associated with larger reductions), consistent with positive changes being related to engagement with ApBM training.

We also explored participants’ reaction time (RT) and error rate data from their ApBM sessions to inform further refinement of the technical parameters of the app.

## Methods

### Design

This was a single-group, open-label, feasibility study. Analyses of drinking, craving, and dependence severity used a repeated measures design.

### Participants

Participants were recruited from across Australia using advertisements on Facebook that directed them to a screening questionnaire hosted on Qualtrics. In addition, online and radio promotions referred participants to a website that contained a brief lay description of ApBM and a link to the screening questionnaire. Participants were required to be aged ≥18 years, have an AUDIT score of at least 8, own a recently updated (ie, within the past year) Android or Apple iOS smartphone with an Australian phone number, and express a desire to reduce or cease their drinking.

### Measures

#### Demographic Information

Participants entered their age, gender, and postcode of residence in an online survey hosted on Qualtrics.

#### Alcohol Problem Severity

The AUDIT was included in the baseline survey to measure the severity of alcohol use and related problems during the past year [38]. The SDS was used to measure severity of psychological dependence in the past month [41], with wording slightly modified to enhance its relevance to alcohol, as recommended by Gossop et al [40].

#### Alcohol Craving

The frequency scale of the Craving Experience Questionnaire (CEQ-F) [42] was used to measure past-week frequency of alcohol cravings. The CEQ-F is a 10-item scale, with each item rated on a scale of 0 to 10. The scale can further be broken down into 3 subscales: “intensity,” “imagery,” and “intrusiveness.” In addition to the CEQ-F, we also utilized a single-item visual analogue scale (VAS) to measure current intensity of alcohol craving immediately before and after each ABM session. Participants were asked “How strongly are you craving alcohol right now?” with a line displayed below the question and a slider that they could place between ends anchored with the words “not at all” on the left end and “extremely” on the right. Participants’ placement of the slider was converted to a number ranging from 0 to 100.

#### Alcohol Consumption

At baseline, participants were asked to estimate the number of days on which they consumed alcohol in the past 28 days. In addition, they were asked to use a calendar chart to enter the number of standard drinks consumed on each of the past 7 days, to allow calculation of the total amount of alcohol consumed, and number of days on which any alcohol was consumed in the past week. To maximize accuracy of self-report, an infographic showing how much wine, beer, or spirits corresponds to 1 standard drink (which, in Australia, is defined as 10 g or 12.7 mL of pure alcohol) was displayed with the calendar chart, and this infographic contained a link to the Australian Government’s Department of Health standard drinks guide [43]. This 7-day drinking assessment was repeated at weekly intervals over the course of the intervention to gather complete drinking data for each week of the 4-week intervention period. At 28 days after the end of the 4-week intervention period, participants were again asked to complete the alcohol consumption calendar chart, estimating the number of days on which they consumed alcohol in the past 28 days and the number of standard drinks consumed on each day in the past week.

#### App Acceptability

At the end of the 4-week intervention, participants completed the “functionality,” “aesthetics,” and ”app subjective quality“ subscales of the uMARS [39]. Individual items of the uMARS range from 1 to 5, with 1 corresponding to very negative, 3 corresponding to neutral/indifferent, and 5 corresponding to very positive assessments, and scores for each subscale are calculated from the mean of individual item scores.

### Intervention

Prior to commencing the intervention, participants were prompted to select 6 alcohol-related images that represent the drinks they most frequently consume. Participants could either take photographs using their phone or select images from a library of 72 alcohol-related images chosen to represent a broad range of alcoholic beverages and brands commonly consumed in Australia. Participants were then prompted to “choose 6 images that represent your goals and motivations for reducing drinking. These could be images of family, friends, pets, hobbies, etc. but must not contain alcoholic beverages.” Again, participants could either use photographs from their phone or select images from a library of 72 images representing a range of healthy activities, positive goals, and sources of pleasure (including family or friends enjoying time together; financial success; employment; exercise, sports, and recreational activities; healthy foods; pets; travel and holidays), which did not contain any depiction of alcohol. Images included in the alcohol and positive image libraries were selected in consultation with a focus group of people with lived experience of treatment for alcohol use problems (see the protocol [33] for further details of consumer input into the development of the app). It should be noted that if participants used their own photographs, these images were not uploaded to a SWiPE server. To maintain their privacy, images were stored only on the participant’s phone, and the SWiPE app only used these files locally while the participant was completing a training session.

**Figure 1:**
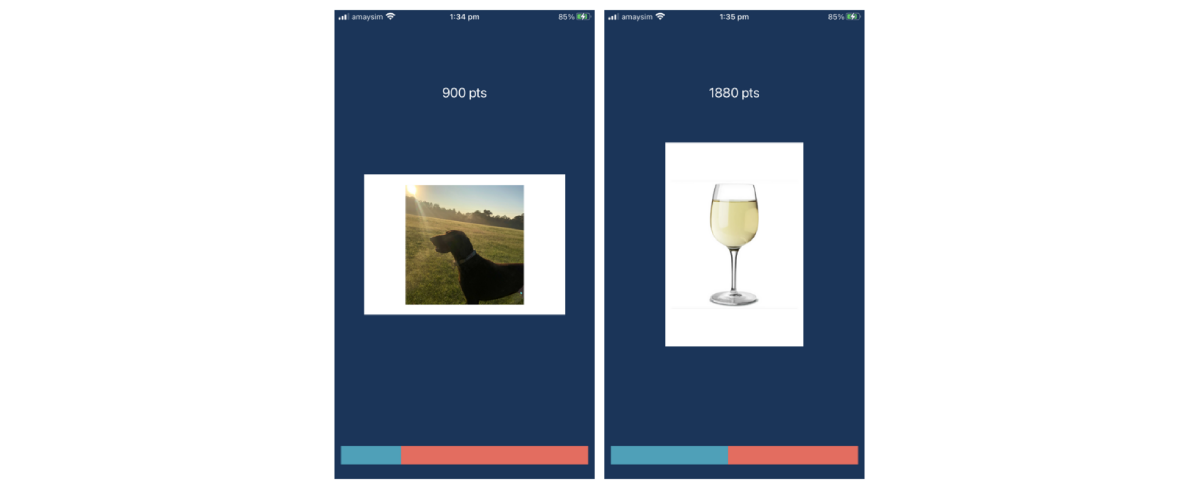
Example of the approach bias modification (ApBM) training task, with approach (left) and avoid (right) stimuli pictured. The user’s score is displayed above each image, and their progress through the ApBM task is displayed in the bar along the bottom of each display.

After selecting their 12 images, participants were presented with instructions for the ApBM task. Images were displayed with a white “frame” around them, which was in either landscape or portrait orientation. When the frame was in landscape orientation, the participant was required to swipe downward (ie, toward themself), which caused the image to expand as if the participant had “pulled” the image “toward” themself. When the frame was in portrait orientation, the participant was instructed to swipe upward (ie, away from themself), which caused the image to shrink until it disappeared as if they had “pushed” it “away.” If the participant swiped in the wrong direction, a red “X” was displayed to inform them that they made an error. Additional technical details regarding image display (including image size, swipe movement criterion, rate of image size change after a swipe response, and interstimulus interval) are reported in the Australian New Zealand Clinical Trials Registry [44]. See Figure 1 for an example of the app’s display during the ApBM task.

Following the display of the instructions, participants completed 10 practice trials (including 5 images in portrait frames and 5 images in landscape frames, in random order) to familiarize them with the task before commencing the first session of ApBM. Each session consisted of 156 trials, comprised of 13 presentations of each image. For each alcohol image, 12 of the 13 presentations were framed in portrait orientation, and 1 presentation was framed in landscape orientation. This was reversed for positive images, whereby 12 of the 13 presentations of each positive image were framed in landscape orientation, while 1 presentation was framed in portrait orientation. Thus, participants were supposed to push away 92.3% of alcohol images and pull 92.3% of positive images toward themselves. If participants made the incorrect response, they were informed that it was an error, but the trial was not repeated.

To increase engagement and encourage participants to respond both quickly and accurately, the task was gamified with a scoring system. Each time the participant swiped an image in the correct direction, they were awarded 10 points. Additionally, they scored “bonus points” for correct responses if their response was fast enough. They received 30 bonus points (yielding a total of 40 points for that trial) if they swiped correctly and within 500 ms of image onset, 20 bonus points (ie, 30 points total) if they swiped correctly within 501 to 1000 ms, and 10 bonus points (ie, 20 points total) if they responded correctly within 1001 to 1500 ms. Correct responses that were slower than 1500 ms following image onset earned only 10 points. If they swiped an image incorrectly (ie, swiped down for portrait or swiped up for landscape), they lost 100 points regardless of their RT. Participants’ scores were displayed on the screen as they performed the task. Upon completion of the task, the final point score was displayed. On the second and subsequent sessions, each participant’s previous session score and the score of their highest-scoring session were displayed prior to commencing the task, to encourage them to beat their previous score.

### Procedure

Individuals interested in participating in the study were directed by social media and online advertising to an online survey hosted by Qualtrics. Study information was displayed along with the option to provide consent to participate. Those who agreed to participate proceeded to a survey that screened for eligibility and collected additional information, including alcohol problem severity and craving (ie, demographic questionnaire, the screening question confirming whether they wished to reduce or cease drinking, AUDIT, SDS, and CEQ-F). Those screened as eligible were required to provide their mobile phone number in order to receive a link via SMS to download SWiPE from the Apple or Google Play Store. Upon first opening SWiPE, they were prompted to provide information about their past-month and past-week alcohol use. Participants were then prompted to select their alcohol-related and positive images and then proceeded to the first session of ApBM. Each session of ApBM was immediately preceded and followed by a VAS craving rating. If a participant’s postsession VAS score was 90 or above after any session, contact details for a national addiction helpline service were displayed.

Participants were prompted by app notifications to complete a minimum of 2 ApBM sessions each week for 4 weeks. In addition, every 7 days, participants were prompted to report the number of standard drinks consumed on each day of the past week. At the end of the 4-week training protocol, participants were prompted to complete a second online survey that included the CEQ-F, SDS, and uMARS. Participants who completed this posttraining survey were given the option to provide their contact details to be in a draw to win 1 of 10 supermarket gift vouchers valued at Aus $100. At 4 weeks after completing training, participants were prompted to complete a final 1-month follow-up questionnaire that assessed past-month and past-week alcohol consumption. Participants were required to complete the follow-up within 48 hours of the prompt being sent for data to be treated as valid. This study was approved by the Monash University Human Research Ethics Committee (project number: 21393).

### Statistical Analysis

The primary outcomes regarding feasibility were the number of sessions completed and the proportion of participants who completed 8 sessions of ApBM within 4 weeks of commencing using the app. The primary outcome for alcohol use was the number of days of alcohol use in the past 7 days (primary time point 4 weeks after commencing the app). Secondary outcomes included uMARS scores (to assess acceptability); number of participants recruited (to assess feasibility); additional alcohol-related outcomes including number of days of alcohol use in the past 28 days, total standard drinks consumed in the past 7 days, SDS score, CEQ-F (and subscale) scores, and single-item craving VAS ratings; and session metrics including trial error rates, RTs, and session durations.

Feasibility and acceptability outcomes, as well as session metrics, were assessed using descriptive data. Changes in alcohol consumption, craving, and SDS scores were analyzed using paired samples *t* tests (in which 2 time points were compared) or repeated measures analyses of variance (RMANOVA) in which 3 or more time points were compared in the same model. To assess possible sources of outcome bias, we conducted analyses comparing characteristics of participants who provided versus those who did not provide outcome data posttraining or at follow-up. These were conducted using independent samples *t* tests for continuous variables (ordinal variables such as days of alcohol use in the past week were treated as continuous since all had at least 8 categories) or Pearson chi-square for categorical variables. To analyze whether number of ApBM sessions moderates the effect of time on past-week drinking and alcohol craving outcomes, we conducted RMANOVA analyses with number of sessions included as a covariate and tested the interaction between number of sessions and time. Descriptive data were analyzed using SPSS version 27 and Microsoft Excel, and inferential analyses were conducted in SPSS version 27. Assuming similar effect sizes for alcohol-related outcomes as that reported by Laurens et al [29] (ie, a 0.36 SD reduction in number of standard drinks per week between pre- and posttraining assessments), we calculated that 119 participants would provide 90% power to detect significant changes using α=.05. As such, we anticipated that the target sample size of 500 would provide ample statistical power to detect main effects of the expected magnitude, even with substantially higher rates of loss to follow-up than anticipated.

## Results

### Recruitment

**Figure 2:**
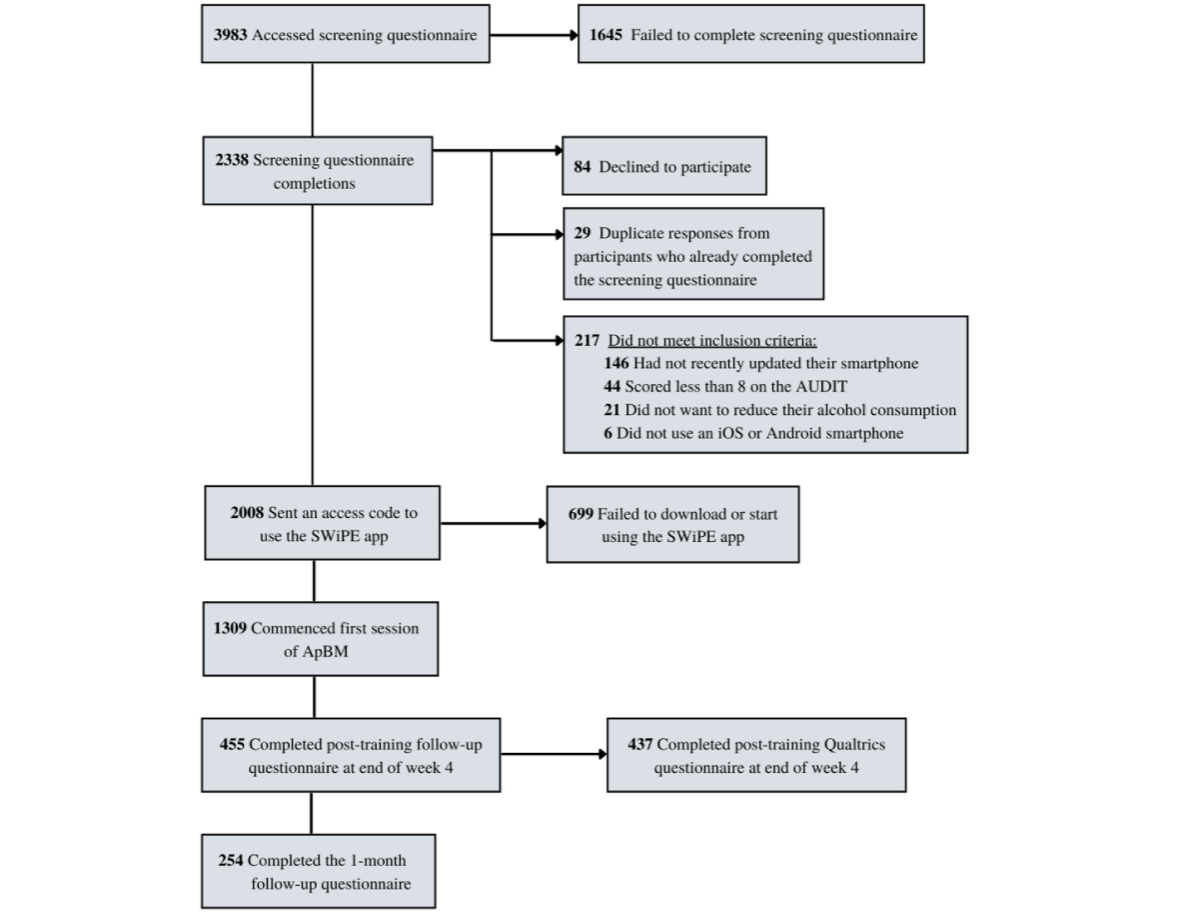
Recruitment and participation flow chart. ApBM: approach bias modification; AUDIT: Alcohol Use Disorders Identification Test.

Recruitment was open for 6 months (August 29, 2020 to February 28, 2021), and during this time, we recruited 1309 participants who met the eligibility criteria, downloaded SWiPE, and commenced at least 1 session of ApBM (see Figure 2).

### Sample Characteristics

Demographic characteristics of the sample are shown in Table 1. The mean age was slightly older, and the sample had a higher proportion of female participants than typical samples recruited from Australian alcohol treatment settings [19,45]. The distribution of the sample between metropolitan, regional, and remote areas corresponded approximately to the Australian general population (of whom 72% live in major cities, 18% in inner regional areas, 8.2% in outer regional areas, 1.2% in remote areas, and 0.8% in very remote areas [46]).

**Table 1:** Demographic characteristics of the sample at baseline (n=1309).

Characteristics		Values
Age (years), range		18-75
Age (years), mean (SD)		47.0 (10.0)
**Gender, n (%)**		
	Female	758 (57.9)
	Male	538 (41.1)
	Other	13 (1.0)
**State/territory^a^, n (%)**		
	Australian Capital Territory	44 (3.4)
	New South Wales	357 (27.4)
	Northern Territory	40 (3.1)
	Queensland	274 (21.0)
	South Australia	89 (6.8)
	Tasmania	49 (3.8)
	Victoria	311 (23.8)
	Western Australia	141 (10.8)
**Remoteness category^b^, n (%)**		
	Major city	864 (66.4)
	Inner regional	293 (22.5)
	Outer regional	128 (9.8)
	Remote	10 (0.8)
	Very remote	6 (0.5)
**Phone type, n (%)**		
	Android	498 (38.0)
	iPhone	811 (62.0)

^a^Data regarding state/territory were missing for 4 participants, and percentages are therefore calculated with a denominator of 1305.

^b^The Australian Bureau of Statistics classifies areas of Australia as “major cities,” “inner regional,” “outer regional,” “remote,” and “very remote” and publishes information regarding which postcodes are located in which remoteness category [46]. Postcode data were missing for 8 participants, and remoteness category percentages were therefore calculated with a denominator of 1301.

The sample’s alcohol use and related characteristics at baseline are shown in Table 2. Despite being recruited from the general community, with the large majority of participants not being in treatment, several indicators suggested high severity of alcohol use and AUD. Both mean AUDIT and SDS scores were above cut-offs that indicate likely dependence (AUDIT >20 [47]; SDS >3 [48]). Indeed, 59.4% (778/1309) of participants scored at least 20 on the AUDIT, and 98.2% (1284/1309) scored at least 3 on the SDS. Participants’ mean alcohol consumption in the week prior to commencing ApBM was nearly 4 times higher than the 10 standard drink per week limit recommended by the National Health and Medical Research Council for minimizing long-term risk of alcohol-related disease [49].

**Table 2:** Alcohol use, dependence, treatment, and craving at baseline (n=1309).

Variable			Values
Number of drinking days (past week), range			0-7
Number of drinking days (past week), mean (SD)			5.3 (1.9)
Number of drinking days (past 28 days), range			0-28
Number of drinking days (past 28 days), mean (SD)			20.7 (6.7)
Number of standard drinks (past week), range			0-221
Number of standard drinks (past week), mean (SD)			37.4 (24.2)
AUDIT^a^ score, range			8-40
AUDIT score, mean (SD)			21.2 (6.5)
SDS^b^ score^c^, range			0-15
SDS score, mean (SD)			7.9 (3.0)
CEQ-F^d^ score^c^, range			0.2-9.9
CEQ-F score, mean (SD)			4.4 (2.0)
**Currently accessing treatment for AUD^e^, n (%)**			
	Yes	117 (8.9)	
	No	1192 (91.1)	
**Alcohol goal, n (%)**			
	Reduce drinking	1102 (84.2)	
	Cease drinking completely	207 (15.8)	

^a^AUDIT: Alcohol Use Disorders Identification Test.

^b^SDS: Severity of Dependence Scale.

^c^Due to missing data, SDS and CEQ-F score statistics come from 1307 participants.

^d^CEQ-F: Craving Experience Questionnaire frequency scale.

^e^AUD: alcohol use disorder.

### Feasibility

The target sample size of 500 participants was recruited within the first 26 days, 7 times faster than anticipated by our hypothesis of 500 recruits within 6 months. Participants completed between 1 and 27 sessions (median 5, IQR 2-9). Participants completed 98.6% (7632/7744) of sessions that were commenced (ie, only 1.4% of sessions that were commenced were not completed), indicating that participants were able to complete sessions without disruption. However, contrary to our hypothesis that at least 60% would complete the 8 sessions, this was only the case for 31.2% (409/1309) of participants.

Participants’ mean number of errors per session was 3.9 (ie, 2.5% of the 156 trials per session), although this was highly skewed (SD 5.5), with the median number of errors per session being 2.25 (1.4% of trials); 95.0% (1244/1309) of participants averaged less than 11.5 errors per session (ie, an average error rate of less than 7.4 of the 156 trials delivered per session).

Analysis of RTs was conducted, excluding participants with average RTs over 3 seconds as these data are likely to be polluted by trials where the participant was distracted from the task for long periods of time (eg, left the phone unattended part way through the session) or repeatedly distracted over many trials. This resulted in exclusion of data for 0.8% (11/1309) of the participants for alcohol trials and 1.4% (18/1309) of the participants for positive trials. The mean of the participants’ average RT was 816.3 (SD 173.3) ms for alcohol trials and 849.3 (SD 203.2) ms for positive images. Examining RTs averaged over both alcohol and positive trials for participants with valid data for both categories (n=1282), only 1 participant (0.1%) achieved a mean RT within the highest reward category (RT<500 ms), 1099 (85.7%) averaged an RT in the second-highest reward category (500<RT<1000), 272 (21.2%) averaged an RT within the third reward category (1000<RT<1500), and 10 (0.8%) had an average RT in the range that did not yield reward points (RT>1500).

### Acceptability

Mean uMARS scores were 4.4 (SD 0.5) for functionality, 4.2 (SD 0.5) for aesthetics, and 3.4 (SD 0.8) for subjective quality. Thus, mean scores were above 3 for all subscales, suggesting generally positive assessments of SWiPE’s acceptability among participants who completed posttraining ratings. Indeed, of 429 participants completing uMARS ratings, 417 (97.2%) gave scores greater than 3 on the functionality subscale, 414 (96.5%) gave scores greater than 3 on the aesthetics subscale, and 293 (68.3%) gave scores greater than 3 on the subjective quality subscale.

### Preliminary Effectiveness

#### Past-Week Drinking Days

**Figure 3:**
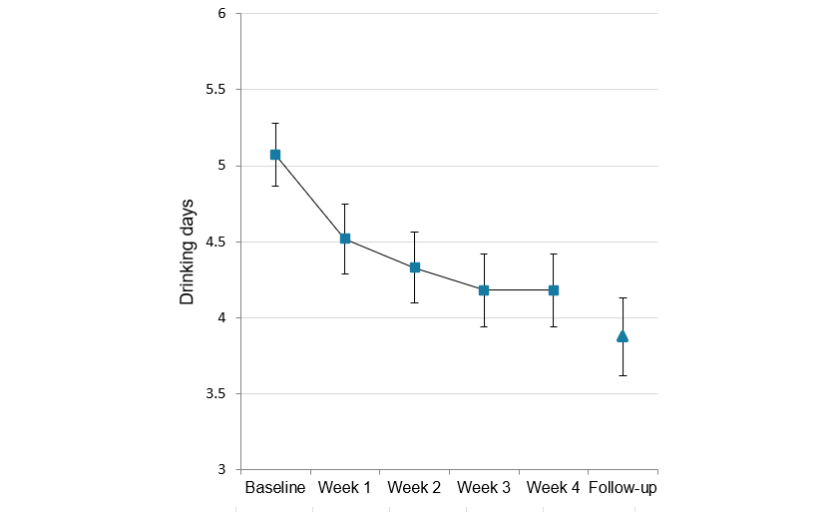
Mean past-week days of alcohol use at baseline and during each week of the intervention for participants with complete data for all 5 time points (n=359) and the 252 who completed the 1-month follow-up. Error bars show 95% CIs of the mean.

Mean number of past-week drinking days at baseline and in each of weeks 1 through 4 among those with complete data for all time points (n=359) is shown in Figure 3. Tests of within-subjects contrasts showed a significant linear effect of time (*F*_1,358_=57.39; *P*<.001; η^2^_p_=0.14), indicating that drinking days tended to decrease over time. The quadratic effect of time was also significant (*F*_1,358_=18.86; *P*<.001; η^2^_p_=0.05), consistent with the smaller week-to-week reductions in drinking days with increasing time apparent in Figure 3. Bonferroni-adjusted pairwise comparisons between weeks showed that drinking days were significantly lower in all weeks of the intervention than they were at baseline (all *P*<.001). Additionally, mean drinking days in week 3 (*P*=.002) and week 4 (*P=*.01) were lower than in week 1 of training. As week 4 was the primary outcome time point, a supplementary paired samples *t* test was conducted comparing baseline (mean 5.1, SD 2.0 days) to week 4 (mean 4.2, SD 2.3 days) in all participants who provided data at both of these time points (n=455). This 18% reduction in weekly drinking days confirmed a robust reduction in the frequency of use (*t*_454_=7.87; *P*<.001; Cohen *d*=0.37). In addition, 9.7% (44/455) of participants reported no alcohol days in the final week of training. Paired *t* tests conducted with participants in the “likely alcohol-dependent” range based on AUDIT score (>20) and who were not receiving treatment indicated that drinking days reduced significantly between baseline (n=207; mean 5.3, SD 1.9) and week 4 (mean 4.4, SD 2.2; *t*_206_=5.82; *P*<.001; Cohen *d*=0.40) and between baseline (n=112; mean 5.4, SD 1.8) and the 1-month follow-up (mean 3.9, SD 2.7; *t*_111_=5.94; *P*<.001; Cohen *d*=0.56).

#### Past-Week Standard Drinks

**Figure 4:**
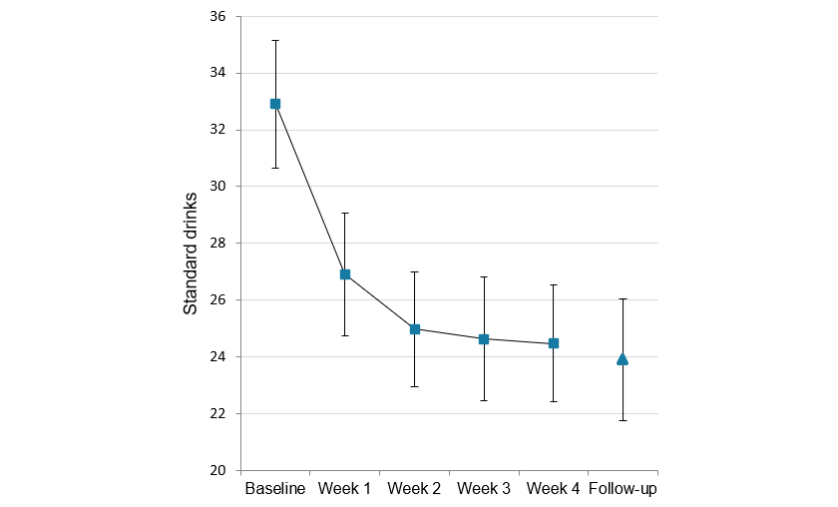
Mean number of past-week standard drinks at baseline and during each week of the intervention for participants with complete data for all 5 time points (n=359) and the 252 who completed the 1-month follow-up. Error bars show 95% CIs of the mean.

Mean number of past-week standard drinks at baseline and in each week of the intervention among those with complete data is shown in Figure 4. Tests of within-subjects contrasts showed a significant linear effect of time (*F*_1,358_=64.91; *P*<.001; η^2^_p_=0.15), indicating that alcohol consumption tended to decrease over time. The quadratic effect of time was also significant (*F*_1,358_=30.8; *P*<.001; η^2^_p_=0.08), consistent with a deceleration in change over time. Bonferroni-adjusted pairwise comparisons between weeks showed that the number of standard drinks consumed was significantly lower in all weeks of the intervention than at baseline (all *P*<.001). Additionally, mean standard drinks in week 3 (*P*=.03) and week 4 (*P=*.03) were lower than in week 1 of training. A supplementary paired samples *t* test comparing baseline (mean 32.8, SD 22.1 standard drinks) to week 4 (mean 24.7, SD 20.1 standard drinks) in participants who provided data at both of these time points confirmed a robust decrease in weekly alcohol consumption by an average of 25% (*t*_454_=8.58; *P*<.001; Cohen *d*=0.40). Among participants in the likely alcohol-dependent range (ie, AUDIT score >20) who were not currently receiving treatment, paired *t* tests indicated that standard drinks reduced significantly between baseline (n=207; mean 41.7, SD 24.2) and week 4 (mean 30.8, SD 22.1; *t*_206_=6.79; *P*<.001; Cohen *d*=0.47) and between baseline (n=111; mean 39.0, SD 18.8) and the 1-month follow-up (mean 30.2, SD 23.4; *t*_110_=3.80; *P*<.001; Cohen *d*=0.36).

### Additional Secondary Alcohol-Related Outcomes

**Figure 5:**
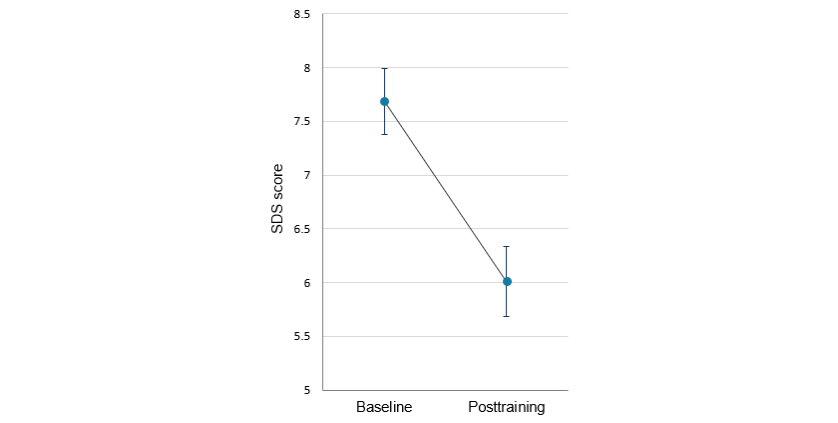
Mean Severity of Dependence Scale (SDS) scores at baseline and posttraining in participants with complete data at both time points (n=436). Error bars show 95% CIs of the mean.

**Figure 6:**
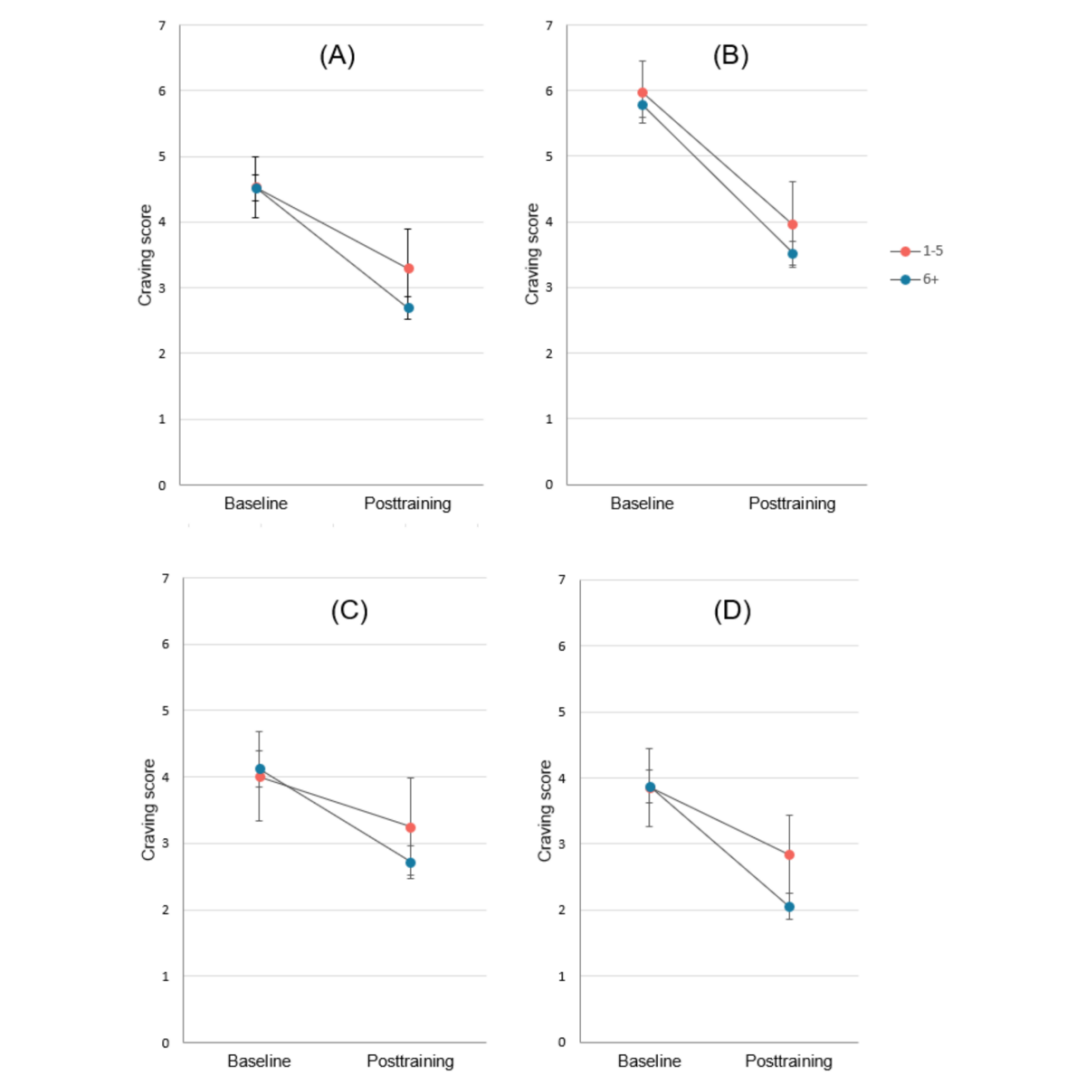
Mean Craving Experience Questionnaire – Frequency Scale (CEQ-F) scores at baseline and posttraining for participants with complete data for both time points who completed 1 to 5 sessions (n=50) and those who completed 6 or more sessions (n=386): (A) total, (B) intensity subscale, (C) intrusiveness subscale, (D) imagery subscale. Error bars show 95% CIs of the mean.

Among participants with complete data for drinking days over the 4-week intervention period (n=359), mean past-month drinking days declined from 20.4 (SD 6.6) to 17.2 (SD 8.1; *t*_358_=8.84; *P*<.001; Cohen *d*=0.47). Participants who completed the SDS at both time points showed a reduction in mean scores from 7.7 (SD 3.0) to 6.0 (SD 3.2; *t*_435_=12.44; *P*<.001; Cohen *d*=0.60; see Figure 5). Mean CEQ-F total scores declined significantly from 4.5 (SD 2.0) to 2.8 (SD 1.8; *t*_435_=19.4; *P*<.001; Cohen *d*=0.93; see Figure 6). Reductions were also significant for all CEQ-F subscales (intensity: *t*_435_=23.2; *P*<.001; Cohen *d*=1.11; imagery: *t*_435_=15.3; *P*<.001; Cohen *d*=0.73; intrusiveness: *t*_435_=11.1; *P*<.001; Cohen *d*=0.53).

**Figure 7:**
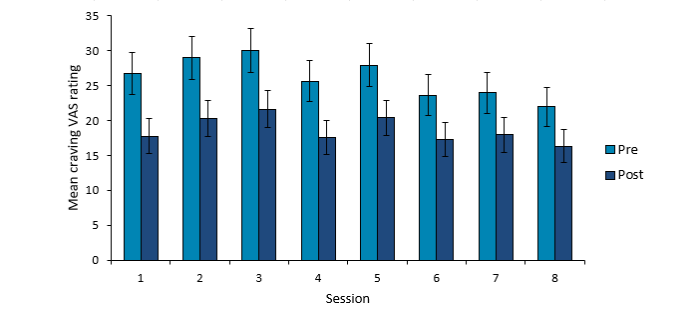
Mean craving visual analogue scale (VAS) ratings before and after each of the first 8 sessions of training among participants with complete data for all sessions (n=380). Error bars show 95% CIs of the mean.

Further evidence for declines in craving come from the single-item craving VAS that was administered before and after each session. Analysis of these ratings across the first 8 sessions among 380 participants with complete data, with session (1, 2, 3, 4, 5, 6, 7, and 8) and timing (presession vs postsession) as separate within-subjects factors, showed main effects for both factors, as well as a significant interaction. Figure 7 suggests that the significant linear effect of session (*F*_1,379_=10.41; *P*=.001; η^2^_p_=0.03) results from a slight tendency for craving ratings to decline in later sessions, relative to earlier ones. There was also a significant quadratic effect of session (*F*_1,379_=5.54; *P*=.02; η^2^_p_=0.01), perhaps reflecting the tendency for craving ratings to increase over the first few sessions, before then declining. As is highly evident in Figure 7, the strong effect of timing (*F*_1,379_=295.93; *P*<.001; η^2^_p_=0.44) reflects mean craving ratings being lower posttraining relative to pretraining across all sessions. This effect of timing significantly interacted with the linear effect of session (*F*_1,379_=27.07; *P*<.001; η^2^_p_=0.07). Separate analyses of presession and postsession ratings suggested that this interaction was due to the linear effect of session being larger for presession ratings (*F*_1,418_=21.16; *P*<.001; η^2^_p_=0.05) than for postsession ratings (*F*_1,379_=4.23; *P*=.04; η^2^_p_=0.01), likely due to a combination of presession craving declining over time and floor effects for the even lower postsession ratings.

### Moderation of Posttraining Outcomes by Number of Completed Sessions

To test whether changes between baseline and week 4/posttraining past-week alcohol use and cravings were associated with the number of sessions completed, we conducted additional RMANOVA analyses in which the interaction term between time (baseline vs week 4/posttraining) and sessions completed was included in the model. Tests of the interaction term indicated that number of sessions completed did not significantly moderate the effect of time on number of past-week drinking days (*F*_1,453_=1.33; *P*=.25; η^2^_p_=0.003) or past-week standard drinks (*F*_1,453_=1.23; *P*=.27; η^2^_p_=0.003). However, it significantly moderated CEQ-F total (*F*_1,434_=8.97; *P*=.003; η^2^_p_=0.02). To better understand this interaction, we classified participants based on whether they completed 1 to 5 or ≥6 sessions (since 5 sessions was the median number completed in the whole sample and 6 sessions is a typical intervention in residential alcohol treatment settings) and tested a RMANOVA with this binary categorization of sessions completed as a between-groups factor. This showed a significant interaction between completion of 6 sessions and time (*F*_1,434_=4.32; *P*=.04; η^2^_p_=0.01), which is depicted in Figure 6. Analyses of CEQ-F subscales suggested this interaction was present for the imagery (*F*_1,434_=6.02; *P*=.01; η^2^_p_=0.01) and intrusiveness: (*F*_1,434_=9.91; *P*=.002; η^2^_p_=0.02) subscales but was not significant for the intensity subscale (*F*_1,434_=2.70; *P*=.10; η^2^_p_=0.006).

### Alcohol Use at Follow-up

**Figure 8:**
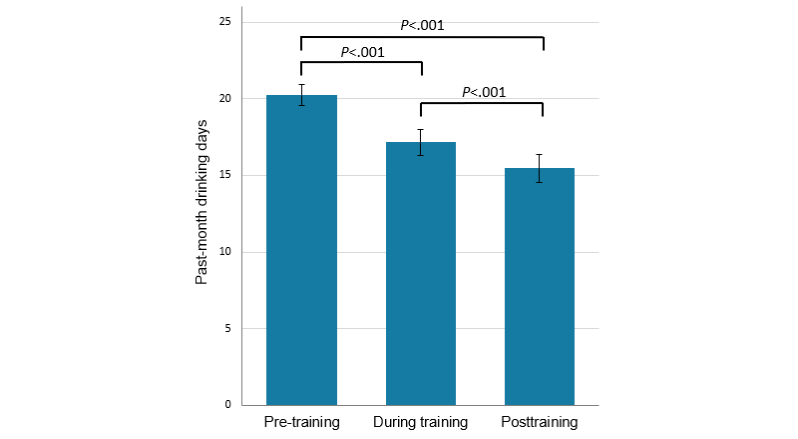
Mean past-month days of alcohol use in the 28 days before, during, and after the intervention for participants with complete data for all 3 time points (n=199). Error bars show 95% CIs of the mean, and horizontal bars display significant Bonferroni-adjusted pairwise comparisons.

The 1-month follow-up survey was completed by 254 participants at 28 to 30 days after the end of the 4-week intervention period (252 of whom provided data regarding past-week and past-month drinking days and 251 of whom provided data regarding past-week standard drinks). Mean drinking days at follow-up were 3.9 (SD 2.5) in the past week and 15.9 (SD 8.8) across the previous 4 weeks. Mean standard drinks consumed in the past week were 23.9 (SD 20.7). Of the 254 participants, 26 participants (10.4%) reported no alcohol use in the past week, and 19 (7.5%) reported no alcohol use in the past month. All alcohol consumption outcomes represented significant reductions relative to baseline (past-week drinking days: *t*_251_=8.07; *P*<.001; Cohen *d*=0.51; past-month drinking days: *t*_251_=8.45; *P*<.001; Cohen *d*=0.53; past-week standard drinks: *t*_250_=5.86; *P*<.001; Cohen *d*=0.37). Interestingly, for participants with complete week 4 and follow-up data, past-week (*t*_227_=3.38; *P*<.001; Cohen *d*=0.22) and past-month drinking days (*t*_198_=4.69; *P*<.001; Cohen *d*=0.33) were also lower at follow-up than in week 4, suggesting continued improvements in the month following the end of the intervention, although number of past-week standard drinks was not reduced between these time points (*t*_227_=–0.14; *P*=.89; Cohen *d*=–0.009). The mean numbers of past-month drinking days before, during, and after the intervention among those with complete data for each time point (n=199) are shown in Figure 8. Tests of within-subjects contrasts showed a significant and strong linear effect of time (*F*_1,198_=66.12; *P*<.001; η^2^_p_=0.25), indicating that drinking days decreased during and after training. The quadratic effect of time was also significant (*F*_1,198_=4.73; *P*=.031; η^2^_p_=0.023). Bonferroni-adjusted pairwise comparisons between the 3 time points showed that drinking days were significantly lower during and after training, compared with baseline, and that drinking days also significantly decreased during training compared with after training (all *P*<.001; see Figure 8). The reductions between baseline and follow-up in past-week drinking days (*F*_1,250_=0.59; *P*=.44; η^2^_p_=0.002), past-month drinking days (*F*_1,250_=0.01; *P*=.91; η^2^_p_<0.001), or past-week standard drinks (*F*_1,249_=0.43; *P*=.51; η^2^_p_=0.002) were not significantly moderated by number of sessions completed.

### Differences Between Posttraining Assessment Completers and Those Lost to Follow-up

Participants (455/1309, 34.8%) completed week 4 assessments of past-week alcohol use within the app, and 33.4% (437/1309) responded to the invitation to complete posttraining questionnaires (SDS, CEQ-F, and uMARS) in the online survey. Participants who completed week 4 alcohol use assessments within the app had a significantly higher mean age than those who did not (49.0 years vs 45.9 years; *t*_1307_=–5.42; *P*<.001) and had completed a substantially higher mean number of ApBM sessions (9.7 vs 3.8; *t*_1307_=–33.96; *P*<.001). These participants generally showed less severe alcohol use in terms of mean AUDIT scores (20.4 vs 21.6; *t*_1307_=3.37; *P*<.001), SDS scores (7.6 vs 8.0; t_1306_=2.40; *P*=.02), past-week drinking days (5.1 vs 5.4; *t*_1307_=3.71; *P*<.001), and past-week standard drinks (32.8 vs 39.8; *t*_1307_=4.98; *P*<.001). In addition, those who provided 4-week outcome data within the app were more likely to use an Android phone (197/455, 43.3% vs 301/854, 35.2%; χ^2^_1_=8.18; *P*=.004). Participants who completed week 4 alcohol use assessments within the app did not significantly differ from noncompleters in terms of gender, remoteness, past-month drinking days, CEQ-F scores, proportion who wanted to reduce versus cease drinking, or proportion currently attending treatment for AUD (data not shown). Comparisons of those who completed the online questionnaires to those who did not generally revealed the same pattern in terms of which differences were significant, with the exception that SDS score and phone type did not significantly differ in this comparison (data not shown).

Comparisons of those who completed the 1-month follow-up revealed a similar pattern, in that they had an older mean age (47.6 years vs 46.2 years; *t*_1307_=–2.08; *P*=.04), had completed a substantially higher mean number of ApBM sessions (10.3 vs. 4.8; *t*_1307_=–22.26; *P*<.001), and tended to drink fewer standard drinks in the week before baseline (32.2 vs 38.6; *t*_1307_=3.81; *P*<.001). They were also more likely than those lost to follow-up to use an Android phone (118/254, 46.5% vs 380/1055, 36.0%; χ^2^_1_=9.46; *P*=.002). However, they did not differ significantly from those lost to follow-up in terms of gender; remoteness category; whether they wanted to reduce or cease drinking; whether or not they were in treatment at baseline; baseline AUDIT, SDS, or CEQ-F total score; or past-week or past-month drinking days (data not shown).

## Discussion

### Principal Findings

This study is the first to examine the feasibility, acceptability, and effectiveness of a personalized, gamified ApBM smartphone app. We found support for the preliminary effectiveness of SWiPE, where participants significantly reduced their standard drinks, drinking days, cravings, and dependence severity at the end of the 4-week training period. Reductions in drinking days and standard drinks not only were maintained during the month after training but also slightly improved even further over time. The results also supported SWiPE’s acceptability, with large majorities of participants providing “positive” scores on the uMARS. Feasibility of SWiPE’s potential for implementation, either in further trials or practice, was strongly supported by the fact that we reached our target sample size within 1 month of commencing recruitment. However, only 31.2% of the sample completed the recommended minimum of 8 sessions. We discuss these findings in the context of the broader literature in the following sections.

### Feasibility

Hypothesis 1 was partially accepted in that we exceeded our recruitment target within 26 days, supporting the feasibility of smartphone-delivered ApBM. Laurens et al [29] reported similar findings, recruiting their sample within 13 days, while Crane et al [27] achieved their recruitment target in less than 2 months. These findings highlight the widespread demand for alcohol reduction apps and the eagerness for people drinking at hazardous levels to try novel approaches such as ApBM. We also found strong evidence for the feasibility of the task itself, with participants completing 98.6% of the sessions they commenced, with a median error rate of 1.4%, which suggests that the task was not overly burdensome, fatiguing, or difficult. However, hypothesis 1 was partially rejected in that fewer than 60% completed the recommended 8 sessions, suggesting the intended “training dose” was not feasible for a large majority of participants in its current form (ie, without additional features or incentives).

### Acceptability

Hypothesis 2, which concerned SWiPE’s acceptability, was accepted in that mean scores on the uMARS subscales were above 3, indicating “good” for the Functionality and Aesthetics subscales and “acceptable” for the Subjective Quality subscale. Laurens et al [29] reported that users rated their ApBM smartphone app—“Breindebaas”—as moderately satisfactory on the Client Satisfaction Questionnaire (mean score of 20.9 out of 30); their users criticized the lack of personalization and game elements in the task. Interestingly, free-text user feedback from our posttest completers praised the options for personalization of stimuli in the ApBM task (particularly the option to use photos from one’s phone library to represent positive values or goals, such as family, friends, and holiday destinations). Given the overall high performance of participants, with most (86%) having mean RTs within the second-highest reward category (501-1000 ms), making the task more challenging may even increase engagement further, though dynamically adjusting to the individual participant’s performance may be optimal, and, in this regard, we recommend further exploration of adaptive difficulty paradigms in the future development of gamified ApBM smartphone applications. Nonetheless*,* based on the uMARS findings and free-text comments, we would recommend that future studies include options for personalization of stimuli and engaging gamification paradigms in order to increase acceptability of ApBM smartphone apps.

### Preliminary Effectiveness

Hypothesis 3 was accepted as there were significant reductions in standard drinks, drinking days, craving, and severity of dependence. The reductions in frequency and quantity of alcohol consumption are consistent with those reported for the Breindebaas app by Laurens et al [29], where participants were encouraged to complete 2 ApBM sessions for 3 weeks leading to an almost identical effect size for reductions in past-week standard drinks. Taken together, these findings suggest that smartphone-delivered ApBM holds promise. However, since controlled trials of delivering ApBM online have found equivalent reductions in active ApBM when compared with sham training and the only prior RCT of an alcohol-reduction app that included ApBM reported null findings [28], it remains necessary to establish the efficacy of smartphone-delivered ApBM in RCTs. This is a particularly worthwhile investment given the greater convenience, flexibility, and accessibility (eg, notification reminders and immediacy of access) that can be offered via smartphones relative to web-based platforms (eg, via a PC or laptop).

Hypothesis 4 was partially accepted. Although there was no clear association between “training dose” (number of sessions completed) and the degree to which participants’ alcohol use was reduced, SWiPE was associated with a reduction in craving, both in the short-term (ie, immediately after a session) and over the duration of the training program. Additionally, it was notable that the effect size (Cohen *d*) for change in craving between baseline and week 4 was much larger than for other outcomes. Interestingly, we also observed a significant moderation effect of number of training sessions on reductions in total CEQ-F score as well as the imagery and intrusiveness subscales. The imagery subscale requires participants to imagine alcohol’s taste, smell, sensation, and how they would picture it, and the intrusiveness scale requires them to reflect on how difficult it is to avoid thinking about alcohol (eg, “how often was it hard to think about anything else”). This is perhaps unsurprising given that SWiPE requires the user to repeatedly view the drinks that they personally regularly consume and practice avoiding them. However, it is important to acknowledge that greater craving reductions in heavier users of SWiPE could also reflect a greater motivation or commitment to reducing one’s alcohol use rather than the “dose” of ApBM itself. Nonetheless, this observed association combined with the significant reduction in VAS craving scores immediately after each session and over time suggests SWiPE may be effective at reducing craving. We also observed significant reductions in participants’ severity of alcohol dependence, which is encouraging given the high proportion in the dependent range on the SDS (98%) and AUDIT (60%) on study entry, despite our intention to recruit participants in the hazardous or harmful drinking range.

### Limitations

These promising results on the preliminary effectiveness and acceptability of SWiPE must be interpreted in the context of the study design and limitations. Although we exceeded our recruitment target, only 33.4% completed the posttraining assessment, and 19.4% completed the 1-month survey. High attrition rates are common in mobile health (mHealth) intervention research, particularly in the absence of monetary incentivization for follow-up completion. The app included prompts (app notifications) to remind participants to complete assessments, yet the rate of participants providing primary outcome data in our study was similar to the 27% in the study by Crane et al [27] and 38% in the study by Laurens et al [29]. As such, it is important to acknowledge the potential attrition bias and overinflation of positive outcomes. It is possible that those who completed follow-ups were more committed to reduce their alcohol use and therefore both engaged more with SWiPE (which we observed) and achieved greater reductions in alcohol consumption, craving, and dependence severity. For similar reasons, the acceptability ratings of the task may be biased toward painting a more positive picture (ie, participants who had a positive assessment of the app may have been more likely to remain engaged enough in the study to complete the posttraining acceptability questionnaire). The lower number of training sessions among those who did not complete the posttraining follow-up suggests the outcomes reported may not be entirely representative of the larger population who engaged in SWiPE. Future studies could reduce the risk of bias posed by high attrition rates by offering incentives for completing follow-ups and engaging in more assertive attempts to contact participants whose follow-ups are overdue. The observed reductions in alcohol consumption, severity of dependence, and craving could also be attributed in part to the Hawthorne effect [50], where participants may have reported reductions in alcohol consumption because of their awareness of being observed in the context of a research study [51]. However, we expect that the absence of personally identifiable data and the anonymity afforded by online self-report methods increased the likelihood of accurate reporting [52].

Another limitation is the reliance on self-reported consumption data, which is always likely to have some degree of inaccuracy (eg, due to poor recall). However self-reported alcohol use is the gold standard in mHealth interventions and alcohol intervention research more broadly [53]. In-person biometric measures to confirm self-report were beyond the scope of the current study given its national focus. We modelled the assessments closely on the computerized 7-day timeline follow-back assessment used by Simons et al [54], which showed good concordance with other measures of alcohol use, and our visual display of standard drink equivalents within the app, when reporting consumption, may have improved the accuracy.

Despite the aforementioned limitations, the study findings advance the ApBM literature by being the first ApBM study to personalize the “avoid” images by using those representing participants’ preferred alcoholic beverages and brands and also the first to personalize the “approach” images to reflect personally meaningful or goal-related behaviors (eg, family, hobbies). Although the positive findings could reflect a potential “dual-target” approach (ie, dampening alcohol associations and reinforcing positive ones), future research would benefit from exploring the extent of reduced alcohol approach bias and increased approach bias to positive cues and the degree to which these changes account for reduced alcohol use.

### Conclusion

Evidence of SWiPE’s feasibility, high acceptability ratings, and multiple indicators of its preliminary effectiveness in terms of reduced alcohol consumption, frequency and quantity, dependence score, and craving are encouraging and suggest an RCT is now warranted. When using SWiPE, consumption (drinking days and standard drinks) decreased significantly. As such, SWiPE may be a useful public health tool given the large number of people drinking at risky levels and in line with the prevention paradox [51,55] where the majority of alcohol-related harm can be attributed to this population (owing to the sheer number of them). Nonetheless, this should not detract from the finding that SWiPE could also be a useful intervention for those with more severe alcohol problems (given that significant reductions were reported among those in the likely-dependent range who were not in treatment when using SWiPE). Establishing its efficacy is a critical next step, as its low cost, ease of implementation, high accessibility, and scalability mean SWiPE could address a significant gap between the demand for treatment and the availability of addiction treatment services [6]. Although we aimed to recruit a sample of individuals drinking at hazardous levels, results (on both the AUDIT and SDS) indicated that the majority of the sample were alcohol-dependent, further demonstrating the critical need for treatment interventions that are available outside of treatment services (particularly given that only 8.9% were currently accessing treatment). Importantly, SWiPE extends neuroscience-informed interventions beyond the laboratory and treatment service environment, ensuring ApBM is an accessible, easy-to-use tool for the broader community. SWiPE has the potential to deliver a “just-in-time” intervention during periods of heightened vulnerability (ie, events, days, and times associated with drinking), by reducing the impulsive subconscious drivers of drinking and enabling people to instead make more conscious, goal-aligned decisions around their alcohol use.

## Abbreviations

ApBM: approach bias modification
AUD: alcohol use disorder
AUDIT: Alcohol Use Disorders Identification Test
CEQ-F: Craving Experience Questionnaire – Frequency Scale
mHealth: mobile health
NHMRC: National Health and Medical Research Council
RCT: randomized controlled trial
RMANOVA: repeated measures analysis of variance
RT: reaction time
SDS: Severity of Dependence Scale
uMARS: Mobile Application Rating Scale – User Version
VAS: visual analogue scale
